# Untargeted metabolomics reveals gender- and age- independent metabolic changes of type 1 diabetes in Chinese children

**DOI:** 10.3389/fendo.2022.1037289

**Published:** 2022-12-22

**Authors:** Jianwei Zhang, Wei Wu, Ke Huang, Guanping Dong, Xuefeng Chen, Cuifang Xu, Yan Ni, Junfen Fu

**Affiliations:** ^1^ Department of Endocrinology, The Children’s Hospital, Zhejiang University School of Medicine, National Clinical Research Center for Child Health, Hangzhou, China; ^2^ Department of Paediatrics, Shaoxing Women and Children Hospital, Shaoxing, China

**Keywords:** type 1 diabetes, metabolomics, children, serum, duration

## Abstract

**Introduction:**

Type 1 diabetes (T1D) is a chronic condition associated with multiple complications that substantially affect both the quality of life and the life-span of children. Untargeted Metabolomics has provided new insights into disease pathogenesis and risk assessment.

**Methods:**

In this study, we characterized the serum metabolic profiles of 76 children with T1D and 65 gender- and age- matched healthy controls using gas chromatography coupled with timeof-flight mass spectrometry. In parallel, we comprehensively evaluated the clinical phenome of T1D patients, including routine blood and urine tests, and concentrations of cytokines, hormones, proteins, and trace elements.

**Results:**

A total of 70 differential metabolites covering 11 metabolic pathways associated with T1D were identified, which were mainly carbohydrates, indoles, unsaturated fatty acids, amino acids, and organic acids. Subgroup analysis revealed that the metabolic changes were consistent among pediatric patients at different ages or gender but were closely associated with the duration of the disease.

**Discussion:**

Carbohydrate metabolism, unsaturated fatty acid biosynthesis, and gut microbial metabolism were identified as distinct metabolic features of pediatric T1D. These metabolic changes were also associated with T1D, which may provide important insights into the pathogenesis of the complications associated with diabetes.

## Introduction

Type 1 diabetes mellitus (T1D), a chronic disease caused by destruction of pancreatic cells and decreased insulin secretion, is accompanied by various complications, and has serious effects on the quality of life and life span (1). According to the newly released Diabetes Atlas from the International Diabetes Federation in 2021, a total of 1,211,900 children and adolescents world-wide had T1D (2) and China is ranked 4^th^ globally for the number of children with T1D (www.diabetesatlas.org).

Recent increases in the incidence of T1D in children and adolescents highlight the importance of environmental factors in disease development. Metabolomic analysis serves as an excellent approach to explore the integrated response of an organism toward environmental changes. Previous metabolomic studies have demonstrated the crucial role of metabolic profiling in discovering biomarker discovery that are predictive of disease incidence and development and potentially its pathogenesis (3–8). For example, Balderas et al. compared the urine and serum metabolome of 34 children with diabetes and 15 non-diabetic controls and discovered that children with T1D had altered bile acid profiles (9). Bile acids are absorbed during enterohepatic circulation, and thus alterations in bile acid profiles may reflect the T1D-associated changes in the gut microbiome. Changes in lipids that play a role in cellular signaling and metabolism in the body during progression to T1D were also noted by several lipidomic studies (5, 9–16). Suvitaival et al. (17) concluded that levels of triacylglycerols, phosphatidylcholines, sphingomyelins, and ceramides were reduced in the plasma of TID children before diagnosis. However, most of the previous metabolomics studies have focused on specific varieties of candidate metabolites, thus failing to effectively provide a complete understanding of the metabolic pathogenesis in TID. Furthermore, these studies have been conducted predominantly in Western populations (18). Data from Asian populations are sparse and limited mainly to cross-sectional studies (19, 20). It is well known that T1D in children and adolescents is affected by age and gender. However, current studies have not stratified the metabolomics spectrum of the disease from the perspective of age and gender.

In the present study, we applied an untargeted metabolomics approach to measure the metabolic profiles of pediatric patients with T1D in the Chinese population, as compared to their healthy controls. The untargeted metabolomics approach was performed using gas chromatography coupled with time-of-flight mass spectrometry (GC-TOFMS). In parallel, we comprehensively evaluated the changes of clinical biomarkers of patients with T1D, including metabolic biomarkers from blood biochemistry, inflammatory cytokines, antibodies, immunoglobulins, and trace elements. This study aims to investigate the metabolic phenome of pediatric T1D and its association with the duration of the disease, and validate the consistency of metabolic changes among male and female patients or at different ages.

## Materials and methods

### Study subjects

The study was approved by the Institutional Review Board of The Children’s Hospital of Zhejiang University, School of Medicine (Approval Number: 2016-JRB-018). Written informed consent was obtained from the guardians of all recruited children, and the study was performed in accordance with the principles of the Declaration of Helsinki. A total of 141 participants including 76 T1D patients and 65 healthy controls were enrolled in this study (**Table 1**). Patients were diagnosed with T1D during their stay at the Children’s Hospital of Zhejiang University School of Medicine and were enrolled in the study between 2016 and 2020. The median duration of the disease since diagnosis was 12 months (range: 1–72 months) and based on this, T1D patients were divided into short-term group (<3 months), mid-term group (3–12 months), and long-term group (>12 months), respectively. T1D was diagnosed based on clinical and biochemical features, specifically elevated blood glucose at presentation (a random measurement of > 11.1 mmol/l and/or fasting blood glucose level of > 7.1 mmol/l), and classical symptoms of diabetes. Furthermore, all patients met at least one of the following criteria: 1) diabetic ketoacidosis (DKA); 2) presence of T1D-associated antibodies (glutamic acid decarboxylase, islet antigen 2, islet cell, or insulin autoantibodies); and/or 3) on-going requirement for insulin therapy. Healthy control refers to the group of children who visited the hospital for routine physical examination, had no disease state, and were enrolled on a voluntary basis.

**Table 1:** Baseline characteristics of the study participants.

Name	T1D (n=76)	Control (n=65)	*p*
Age (in months)	109.2 ± 47.07	110.9 ± 41.33	0.1325
Gender			
Male	29 (44.6%)	31 (47.7%)	
Female	47 (55.4%)	34 (52.3%)	
Height, cm	132.1 ± 24.93	131.3 ± 20.75	0.09
Weight, kg	30.0 ± 13.76	29.4 ± 11.58	0.20
BMI, kg/m^2^	16.4 ± 2.69	16.3 ± 1.90	0.14
Duration (in months)	16.7 ± 18.9	—	—

Normally distributed variables were analyzed using student t-test and presented as mean ± standard deviation (SD), while non-normally distributed variables were performed by non-parametric Mann-Whitney U test and presented as medians and interquartile range (25th–75th percentiles). T1D, type 1 diabetes; BMI, body mass index.

### Clinical measurements

The medical records and routine laboratory biochemistry data of the participants were summarized in **Table 2**. Serum lipid profiles (i.e., total cholesterol, HDL-cholesterol, LDL-cholesterol, and triacylglycerols), total protein, apolipoprotein A1, apolipoprotein B, lipoprotein A, carboxyhemoglobin, high-sensitivity C-reactive protein, albumin, globulin, glycated hemoglobin (HbA1C), insulin-like growth factor 1 (IGF-1), insulin-like growth factor-binding protein 3, alanine aminotransferase (ALT), alkaline phosphatase (ALP), aspartate aminotransferase (AST), lactate dehydrogenase (LDH), total bilirubin, direct bilirubin, and indirect bilirubin were measured. Human inflammatory cytokine multiple ELISA kit was used to quantitatively measure cytokine levels including interferon-gamma (IFN-γ), interleukin (IL)-10, IL-2, IL-4, IL-6, and tumor necrosis factor-alpha (TNF-α). Multiple monoclonal antibodies that recognize a common cell-surface antigen are combined to form clusters of differentiation. The clusters are numbered sequentially with respect to when they were discovered and defined. The cell-surface reactivity of monoclonal antibodies to each CD antigen was detected by flow cytometry.

**Table 2:** Characteristics of clinical measurements.

Name	HC Median (reference range)	T1D (Median ± SD)	Median	FC
TSH (uIU/ml)	2.645 (0.35–4.94)	2 ± 1.12	2	0.636
Potassium (mmol/L)	4.5 (3.5–5.5)	4 ± 0.35	4	0.842
Sodium (mmol/L)	140 (135–145)	138 ± 4.94	138	0.987
Glucose (mmol/L)	4.85 (3.6–6.1)	12 ± 5.79	12	2.515
Total Bile Acids (µmol/L)	6.0 (0.0–12.0)	5 ± 22.19	5	0.783
Uric acid (µmol/L)	256 (155–357)	220 ± 90.1	220	0.859
25(OH)D (ng/ml)	88.75 (27.5–150.0)	48 ± 19.31	48	0.544
Hemoglobin (g/L)	140 (120–160)	133 ± 11.68	133	0.951
PLT (10^9^/L)	250 (100–400)	291 ± 82.44	291	1.163
WBC (10^9^/L)	8.0 (4.0–12.0)	7 ± 4.07	7	0.865
Insulin (µg/L)	12.45 (1.9–23)	4 ± 23.88	4	0.321
HOMA-IR (%)		2 ± 14.38	2	
ALT (U/L)	<50	13 ± 10.05	13	
Urea (µmol/L)	4.11 (1.79–6.43)	5 ± 1.7	5	1.128
Cholesterol (mmol/L)	4.35 (3.00–5.70)	4 ± 1.83	4	0.961
CKMB	<25	23 ± 13.36	23	
Creatinine (µmol/L)	46 (15–77)	58 ± 14.44	58	1.261
GGT (U/L)	32.5 (8–57)	12 ± 3.4	12	0.369
TC (mmol/L)	<1.70	1 ± 7.08	1	
HDLC (mmol/L)	>1.04	1 ± 0.4	1	
LDLC (mmol/L)	<3.37	2 ± 1.03	2	
HbA1c (%)	5.4 (4.5–6.3)	8 ± 2.86	8	1.463
HsCRP (mg/L)	4 (0–8)	4 ± 6.36	4	1
β2-MG (mg/L)	0.15 (0.00–0.30)	0 ± 2.45	0	0.715
C3 (g/L)	1 (0.50–1.50)	1 ± 0.26	1	1
C4 (g/L)	0.25 (0.10–0.40)	0 ± 0.1	0	0.8
IgG (g/L)	10.2 (6.36–14.04)	11 ± 2.42	11	1.058
IgM (g/L)	0.75 (0.29–1.21)	1 ± 0.48	1	1.44
Urinary Creatinine (μmol/L)	11275 (2550–20000)	4781 ± 3491.06	4781	0.424
Uridine triphosphate (mg)	<100.0	12 ± 270.56	12	
Urine α1-microglobulin (mg/L)	<12.00	7 ± 13.63	7	

FC is the fold change ratios by calculating the median value of each clinical marker in the T1D group vs. the reference range. HC, healthy control; T1D, type 1 diabetes; FC, fold change; TSH, thyroid stimulating hormone; PLT, total platelet count; WBC, white blood cell count; HOMA-IR, homeostatic model assessment for assessing insulin resistance; ALT, alanine transaminase; CKMB, creatinine kinase myocardial band; GGT, gamma-glutamyl transferase; TC, total count; HDLC, high density lipoprotein cholesterol; LDLC, low density lipoprotein cholesterol; Hb1Ac, hemoglobin A1c; HsCRP, high-sensitivity C-reactive protein; β2-MG, Beta-2 microglobulin; C3, complement component 3; C4, complement component 4; IgG, immunoglobulin G; IgM, immunoglobulin M.

### Metabolomics

Blood samples were collected after fasting overnight for at least 8 hours and centrifuged to obtain serum prior to storage in -80°C freezer. The untargeted metabolomics profiling of serum samples was performed on a GC-TOFMS system (Pegasus BT, Leco Corp., St. Joseph, MO, USA) equipped with an Agilent 7890B gas chromatograph and a Gerstel multipurpose sampler with dual heads (Gerstel, Muehlheim, Germany). The procedure was performed as described in a previously published paper with minor modifications (21). Briefly, each aliquot of 50 µL serum sample was mixed with 10 µL of internal standard, to which 175 µL of pre-chilled methanol/chloroform (v/v=3/1) was added for metabolite extraction. After centrifugation at 13,500 rpm for 20 min at 4°C (Microfuge 20R, Beckman Coulter, Inc., Indianapolis, IN, USA), the supernatant was carefully transferred to an autosampler vial. The samples in autosampler vials were then evaporated briefly to remove chloroform using a CentriVap vacuum concentrator (Labconco, Kansas City, MO, USA), and further lyophilized with a FreeZone freeze dryer equipped with a stopping tray dryer (Labconco, Kansas City, MO, USA). The sample derivatization was performed by a robotic multipurpose sampler with dual heads (Gerstel, Muehlheim, Germany). Specifically, the dried sample was derivatized with 50 µL of methoxyamine (20 mg/mL in pyridine) at 37.5°C for 2 hr., followed by incubation at 37.5°C for 1 hr after the addition of 50 µL of MSTFA (1% TMCS) containing FAMEs as retention indices. Separation and sample derivatization were performed as parallel operations. A Rxi-5 ms capillary column (30 m × 250 µm i.d., 0.25 µm film thickness; Restek corporation, Bellefonte, PA, USA) was used for metabolite separation. The temperature was initially held at 80°C for 2 min, then ramped up to 300°C at the rate of 12°C/min, held for 4.5 min, then further ramped up to 320°C at the rate of 40°C/min, and finally, held for 1 min. Helium was used as the carrier gas at a constant flow rate of 1.0 mL/min. The temperature of the injector and the transfer interface were both set to 270°C and the injection volume was 0.5 µL in spitless mode. Measurements were made using electron impact ionization (70 eV) in the full scan mode (m/z 50–500). Instrument optimization was performed as needed.

### Metabolite annotation

Metabolite annotation was performed by comparing the retention indices and mass spectral data with those previously generated from reference standards in the in-house library (22). The reference chemicals were commercially purchased from Sigma-Aldrich (St. Louis, MO, USA), Santa Cruz (Dallas, TX, USA), and Nu-Chek Prep (Elysian, MN, USA). Commercial libraries such as NIST library 2017 and LECO/Fiehn Metabolomics library for GC-TOFMS were used for cross-validation analysis. The direct relationship of two adjacent metabolites from the known metabolic relation network (KEGG) was used to indicate the alteration of specific metabolic enzyme, thereby providing complementary biological information for metabolite interactions. Metabolites were annotated in the serum samples with those of pure chemical standards. Metabolites that did not pass our QC criteria (CV>20%) were removed from further statistical analysis, as the purpose of this project was to provide data for a further validation study, rather than making a simple biomarker discovery. Missing values were initially imputed using QRILC method reported in our previous work (23).

### Data analysis

The medical records and the routine laboratory biochemistry data were statistically analyzed using R packages ver. 4.0.2. The details of statistical methods applied in this study, R functions and packages were summarized in **Supplementary Table 1**. Specifically, normally distributed variables were analyzed using student *t*-test and presented as mean ± standard deviation (SD), while non-normally distributed variables were performed by non-parametric Mann-Whitney U test and presented as medians and interquartile range (25th–75th percentiles). The raw metabolomic data generated by GC-TOFMS were processed using ADAP software (24). To reduce bias caused by the high blood glucose levels in diabetic patients, glucose was excluded from the final data set. The metabolome data were further standardized before statistical modeling. Unsupervised principal component analysis (PCA) was used to evaluate the natural clustering between patients with T1D and their healthy controls. Each dot represented an individual subject and color-coded based on their grouping. To overcome the complexity of biological samples, a widely-used supervised orthogonal partial least square discriminant analysis (OPLS-DA) model was applied to capture the differential metabolites between the two groups. The OPLS-DA model was constructed using 1/7-fold cross-validation. Metabolic pathway enrichment analysis was done using the hypergeometric algorithm deployed in MetaboAnalyst (25). The significance of the metabolic pathways associated with T1D was determined by the cutoff p-value of 0.10. All the *p* values were adjusted by Benjamini & Hochberg method. Spearman correlation analysis was used to evaluate the correlation between each differential metabolite and disease duration.

## Results

### Clinical characteristics and metabolic profiles of T1D patients

**Figure 1:**
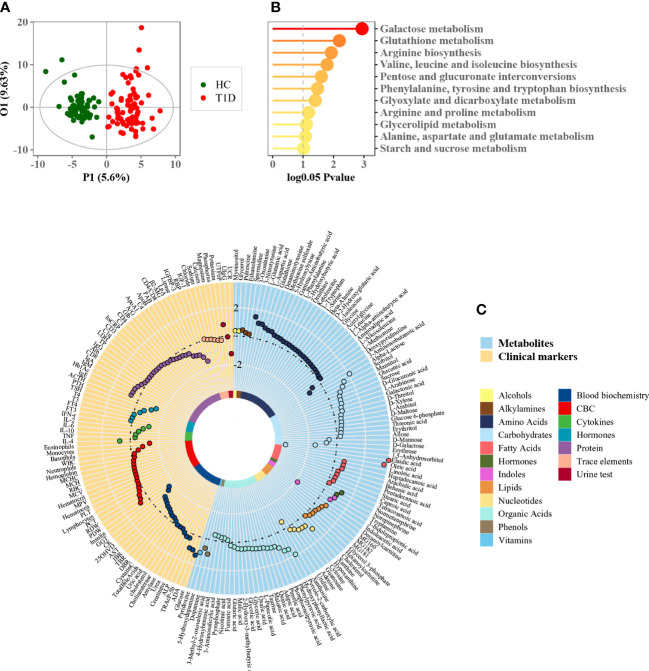
Phenome and metabolome analysis of T1D patients. **(A)** OPLS-DA score plot of patients with T1D and healthy controls (each green dot represents a healthy subject while each red dot denotes a T1D patient). **(B)** Metabolic enrichment pathway analysis using MetaboAnalyst. **(C)** Circle plot of phenome and metabolome fold changes (T1D versus healthy controls). Each dot was color-coded based on their chemical classes or clinical diagnostic purpose.

The basic demographic information of the participants is summarized in **Table 1**. No significant differences were observed in gender, age, and BMI between patients with T1D and healthy controls. Serum lipid profiles are summarized in **Table 2**. The mass spectrometry-based metabolomics study detected 282 circulating metabolites that were present across all the study samples, with a low median process variability among QC samples (<20%). A total of 51 metabolite-metabolite ratios were also calculated according to their metabolic reactions. The identified serum metabolome covered over 60 biochemical pathways of human metabolism and included a wide range of metabolite classes such as amino acids, organic acids, fatty acids, alcohols and sugar derivatives, lipids, nucleotides, indoles, and phenols. The OPLS-DA scores plot depicts the distinct metabolic profiles associated with T1D patients versus healthy controls (**Figure 1A**). A total of 70 differential metabolites and 14 metabolite ratios were obtained between T1D patients and healthy controls with FDR-corrected *p* value (*p* < 0.01). Carbohydrates and organic acids altered apparently, and the most significantly altered metabolites were 1,5-anhydrosorbitol, α-lactose, indole acetic acid, arachidic acid, and so forth. The majority of these metabolites were significant after age- and/or gender adjusted (**Supplemental Table 2**). Based on these differential metabolites, the metabolic enrichment analysis indicated that 11 pathways were significantly perturbed in T1D patients as compared to controls (**Figure 1B**), including glucose metabolism, glutathione metabolism, arginine and proline metabolism, branched chain amino acid (BCAA) metabolism, etc. Among these, galactose metabolism was the most significantly altered metabolic pathway associated with T1D, and specifically increased α-lactose, sorbitol, myoinositol, sucrose, glycerol, and reduced d-mannose and d-galactose were identified in this study. Additionally, we examined the clinical characteristics of T1D patients, including blood biochemistry, complete blood count (CBC), cytokines, hormones, proteins, trace elements, and urine tests. An integrative view of both clinical characteristics and metabolic changes of T1D patients is illustrated in **Figure 1C**. Clinical markers that were different included elevated levels of urine transferrin, IgE, IL-4, tartrate resistant acid phosphatase (TRAcP-5b), adenosine deaminase (ADA), and glucose levels, as well as reduced levels of urine creatinine, IGF-1, adrenocorticotropic hormone (ACTH), IFN-r, eosinophils, insulin, and r-glutamyl-transferase (GGT).

### Association of age and gender factors with circulating metabolome

**Figure 2:**
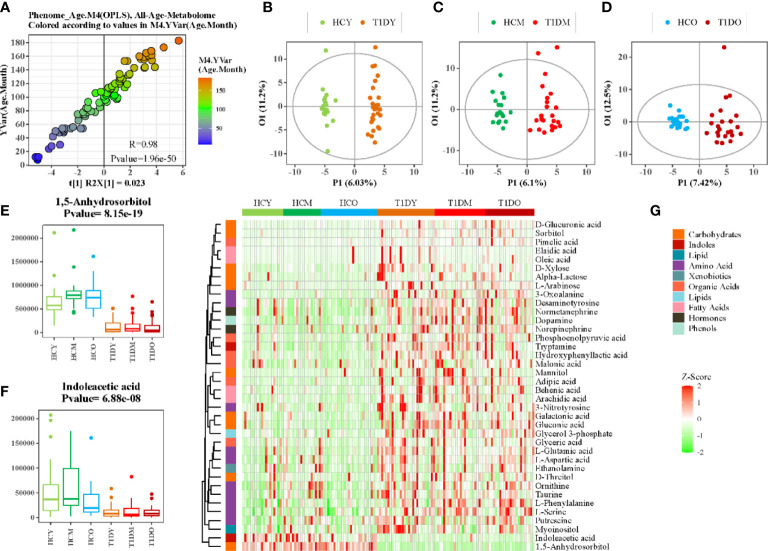
Association analysis between age and serum metabolome. **(A)** Scatter plot of OPLS-DA PC1 scores and ages of patients. **(B–D)** OPLS-DA score plot of patients with T1D and healthy controls at different ages, B:(1-84 months), C:(85-120months), and D:(121-185months). **(E, F)** Box plots showing the concentration of selected metabolites at different ages. **(G)** Heatmap of z-score values for differential metabolites between patients with T1D and healthy controls at different ages.

Although age- matched controls were used in this study, age was believed to be a confounding factor of host metabolism that deserves thorough investigation, particularly for children. The OPLS regression model revealed that metabolic profile variations correlated closely with the age of T1D patients (**Figure 2A**, r = 0.98, *p* = 1.96e-50). The patients were then further stratified into three different subgroups according to their age: young (1–84 months), middle (85–120 months), and old (121–185 months). The baseline characteristics of patients in these three subgroups are shown in **Table 1**. The metabolic profile of patients in each subgroup was compared with age-matched healthy controls, and each comparison consistently showed an apparent separation between the two groups according to the OPLS-DA score plot (**Figures 2B–D**). The heatmap of z-score values derived from each comparison (FDR-corrected *p* value < 0.01) showed the relative expressions of differential metabolites among patients and healthy controls, and indicated whether specific metabolic changes were consistent across different age groups (**Figure 2E**). For example, 1,5-anhydrosorbitol and indoleacetic acid were significantly reduced in T1D patients of all different ages (**Figures 2F, G**).

**Figure 3:**
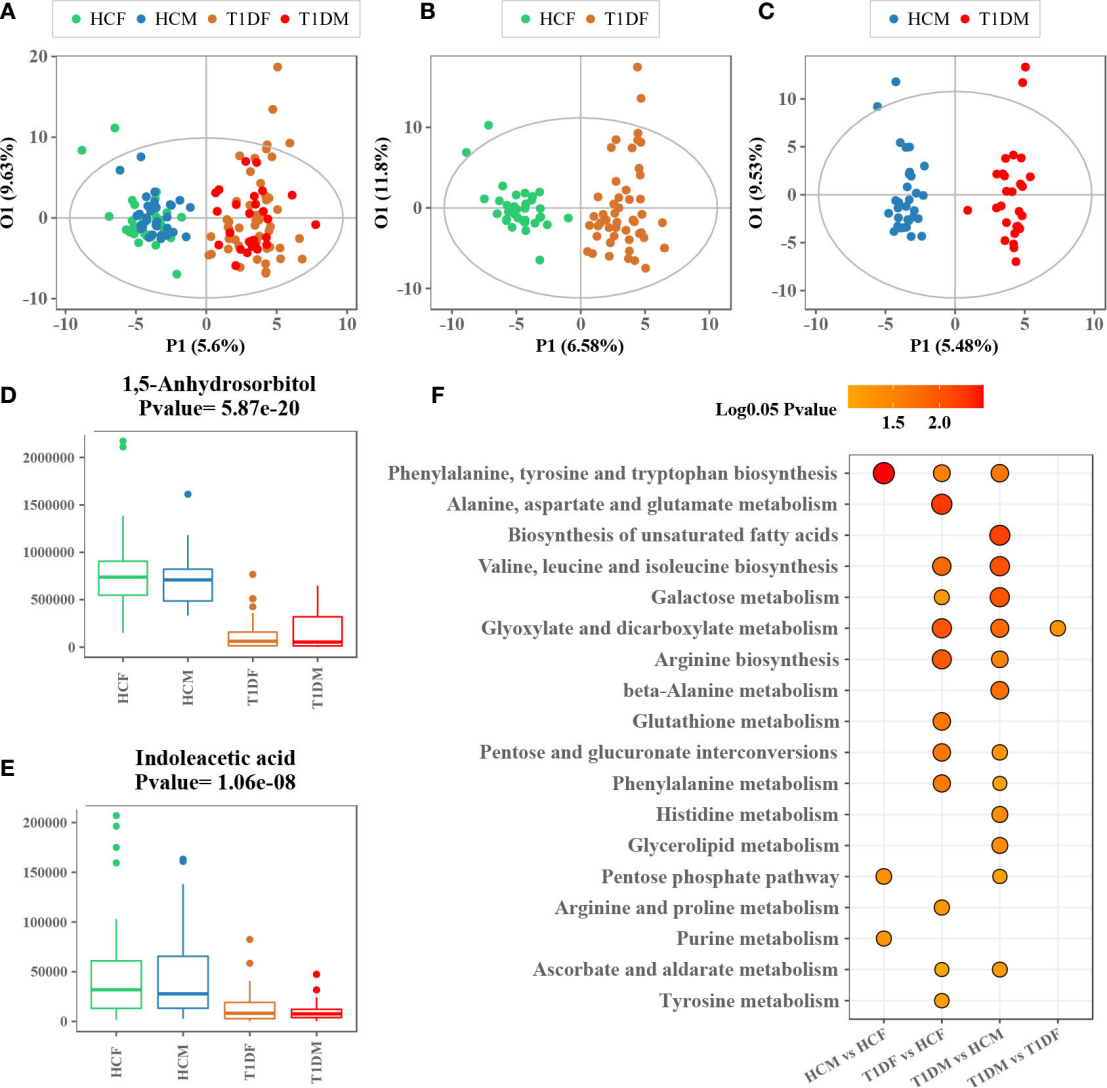
Association analysis between gender and serum metabolome. **(A)** OPLS-DA score plot of patients with T1D and healthy controls labeled with information on gender of the participants. **(B, C)** OPLS-DA score plot of patients with T1D and healthy controls for males and females. **(D, E)** Box plots showing the concentration of representative metabolites across different groups. **(F)** Metabolic enrichment pathway analysis for different group comparisons.

Similarly, we also examined the impact of gender on metabolism. The OPLS-DA model with information on the gender of the participants did not show any obvious internal variations among patients with T1D or healthy controls (**Figure 3A**). Moreover, to eliminate the influence of gender, OPLS-DA model was applied to compare the metabolic variations between male and female T1D patients (29 and 49, respectively) and healthy controls (31 and 34, respectively), separately (**Figures 3B, C**). The differential metabolites, 1,5-anhydrosorbitol and indoleacetic acid, were significantly reduced in both male and female patients with T1D (**Figures 3D, E**). The enrichment pathway analysis based on male and female differential metabolites further validated that most of differential metabolic pathways were consistent in both male and female patients. However, we found that four pathways namely, (i) phenylalanine, tyrosine and tryptophan biosynthesis, (ii) pentose phosphate pathway, (iii) purine metabolism, and (iv) glyoxylate and dicarboxylate metabolism, could be affected by the gender (**Figure 3F**).

### Association of disease duration with circulating metabolome

**Figure 4:**
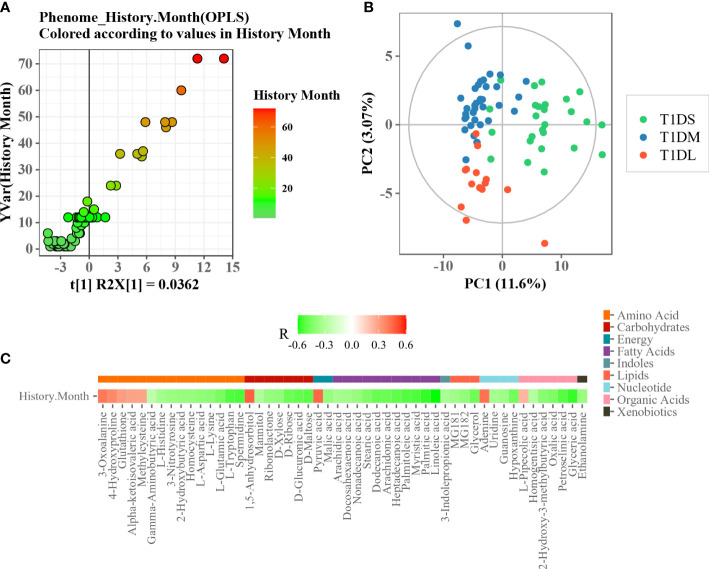
Association analysis between disease duration and serum metabolome. **(A)** Scatter plot of OPLS PC1 scores and disease duration. **(B)** PLS-DA score plot of T1D patients with different disease durations. **(C)** Heatmap of Spearman’s correlation between differential metabolites and disease durations. The metabolite was assigned to each metabolite class (plotted with different color).

To determine whether there is an association of disease duration and serum metabolome, the orthogonal partial least squares regression (PLSR) analysis was performed (**Figure 4A**). This analysis depicted an obvious linearity between phenome and disease duration of T1D patients. The patients were further divided into three major clusters: initial progression from disease onset (short, 1–6 months), moderate phase (medium, 7–18 months), and advanced phase (long, 1.5-6 years). As shown in **Figure 4B**, the metabolic profiles of the three subgroups were clearly separated according to the score plot of PLS-DA model. The heat map indicated a significant Spearman’s correlation between metabolites belonging to nine chemical classes and disease duration (**Figure 4C**). Of them, 1,5-anhydrosorbitol, pyruvic acid, and adenine had the strongest positive correlations with disease duration.

## Discussion

The pathogenesis of diabetes in children is complicated due to the frequent occurrence of future complications. Insulinopenia and hyperglycemia, characteristics of the T1D milieu, profoundly alter metabolic homeostasis. Altered metabolites affected by the variable insulin and glycemic levels may theoretically increase the risk of long-term complications. While diabetes is primarily characterized by hyperglycemia, other nutrient metabolic pathways like amino acid and tricarboxylic acid cycle (TCA) are also profoundly perturbed. However, a comprehensive metabolic signature for T1D, especially in Chinese children, was not previously established. Hence, we performed a serum metabolomics study of Chinese children with T1D. Utilizing both multivariate and univariate statistical analyses, a unique metabolic pattern was observed to be related to T1D. This included 70 differently expressed metabolites that were associated with 11 specific altered metabolic pathways. These metabolic changes were also investigated by sub-dividing the patients based on different ages and gender. In addition, an integrative analysis of clinical features and metabolic profiles was performed, which provided us a comprehensive view of pediatric T1D. An interesting finding was that a group of differential metabolites were closely associated with the time elapsed since diagnosis of the disease, which might provide important insights into the pathogenesis of the complications associated with diabetes.

Dysregulated carbohydrate metabolism was an obvious metabolic feature of T1D observed in this study. Particularly, galactose metabolism was the most significantly altered metabolic pathway associated with T1D (**Figure 1B**). This alteration remained significant across different ages and in both genders of T1D patients compared to healthy controls, but it was similar between young and old participants (**Figure 2G**), or between male and female counterparts (**Figure 3F**). We also observed that glutathione, arginine, and BCAA metabolism were significantly altered between the T1D and control groups. In normal physiological conditions, glutathione has antioxidative and free radical-scavenging roles, thereby maintaining the metabolism and homeostasis of cells (26, 27). Metabolic disorder of the glutathione pathway increases oxidative stress, which may damage kidneys and blood vessels, and cause neurodegeneration. Arginine is the precursor for oxide synthesis, which can be converted to vasodilating factors in the body. On one hand, arginine can stimulate insulin secretion, but on the other hand, arginine produces NO, which is involved in response to oxidative stress in organisms, and participates in glutathione metabolism. Disorders of arginine metabolism may affect endothelial cell function, leading to insulin resistance and disturbances in metabolism and hemodynamics (28). BCAAs include leucine, isoleucine, and valine, which are essential amino acids that provide energy to the body (29). Disordered BCAA metabolites can block insulin signaling and disturb lipid metabolism, resulting in insulin resistance and excessive lipid accumulation, respectively (30). BCAA metabolism disorder is a biomarker of cardiovascular metabolic diseases (31).

Among the differential carbohydrates, 1,5-anhydroglucitol (1,5-AG) was the most significant marker that was consistently lower in T1D patients, regardless of gender or age. Serum 1,5-AG has been considered a potential marker of short-term glycemic control and can be used for T1D diagnosis or the screening of high-risk patients. Moreover, it was found to be less influenced by diet or physical activity as compared to point glycemic markers (32). Thus, 1,5-AG can be an effective supplementary marker to hemoglobin A1c. Low plasma levels of 1,5-AG are associated with decreased pancreatic β-cell function (33). Sorbitol takes part in the polyol pathway through the reduction of intracellular glucose to sorbitol. The polyol pathway gets activated when excess glucose is present within the cells and thus, a hyperglycemic state might accelerate intracellular accumulation of sorbitol. Furthermore, excessive sorbitol in the cells has been associated with a pro-oxidative environment, which is known to increase diabetes-related complications (34, 35). Renal tubular reabsorption of 1,5-AG is inhibited when there is excess glucose in the plasma. Studies have found that 1,5-AG levels decrease in patients with diabetes and this decrease is related to kidney damage owing to high blood glucose levels (36).

We found that compared with the healthy group, TCA cycle metabolites (pyruvate, fumarate, malate, and linoleic acid) were significantly increased in the T1D group. Higher concentrations of pyruvate appear to be necessary for anaplerosis (37). The increased linoleic acid might be related to the insulin resistance in patients with T1D. The increase of fatty acids can lead to the slowing down of the tricarboxylic acid cycle e.g., the accumulation of fumarate and malate, which can block the oxidation of glucose. Some studies have considered that high physiological levels of exogenous insulin and hyperglycemia could be responsible for insulin resistance in T1D patients (38, 39).

In the present study, indoleacetic acid was significantly reduced in the diabetic group, suggesting that T1D was associated with the metabolism of indole and its derivatives. Dietary tryptophan can be metabolized into IAA by gut microbiota through the indole-3-acetamide pathway under the catalysis of tryptophan monooxygenase and indole-3-acetamide hydrolase (40). Recent work has demonstrated that gut microbiota is an essential modulator of T1D susceptibility and the reduced IAA levels were associated with intestinal mucosal barrier integrity impairment (41). Dysfunction of the intestinal mucosal barrier can increase intestinal permeability and trigger an immunological response, contributing to the development of various autoimmune diseases, Including T1D (42). Indole acetic acid is also involved in the metabolism of purines, and this metabolic cycle is closely related to the pathogenesis of diabetic nephropathy.

We also found that adenine, 1,5-AG, and pyruvate correlated strongly and positively with the course of the disease. Clinically, accumulation of adenine in the blood results in insoluble crystal (2, 8-dihydroxyadenine) precipitates in the renal tubules and obstruction of tubular flow, which initiates renal injury (43, 44). A similar disease phenotype can be induced in rodents by adenine-feeding (45, 46). Adenine feeding-induced chronic kidney disease in rodents is characterized by elevated plasma concentrations of urea and creatinine, proteinuria, interstitial fibrosis, extensive tubular dilation, degeneration of the proximal tubular epithelium with loss of the brush border and inflammatory cell infiltration (47, 48).

In summary, metabolomics is a powerful tool for investigation of the nature-nurture relationships involved in the development of pediatric diabetes. Overall, in this study, carbohydrate metabolism, unsaturated fatty acid biosynthesis, and gut microbial metabolism were identified as distinct metabolic features of pediatric T1D. These metabolic changes were also associated with T1D, which may provide important insights into the pathogenesis of the complications associated with diabetes. The limitation of this study is the lack of fecal samples for the analysis of gut microbiome to confirm the role of microbial metabolites in T1D, e.g., IAA metabolism. Further studies are needed to explore the complex relationship between gut microbiome and metabolism in the pathogenesis of T1D. In the future, larger studies are needed to determine whether these metabolic markers can add to the prediction of long-term T1D.

## Data availability statement

The raw data supporting the conclusions of this article will be made available by the authors, without undue reservation.

## Ethics statement

The study was approved by the Institutional Review Board of The Children’s Hospital of Zhejiang University, School of Medicine (Approval Number: 2016-JRB-018). Written informed consent to participate in this study was provided by the participants’ legal guardian/next of kin.

## Author contributions

JF and JZ conceived and designed the study. WW, KH collected the plasma samples. GD and XC provided patient care and were responsible for communication with the parents. YN conducted the metabolomics analysis. YN and CX performed the data analysis. JZ and YN wrote the manuscript. All authors contributed to the article and approved the submitted version.
