# Supplementary material for: Untargeted metabolomics reveals gender- and age- independent metabolic changes of type 1 diabetes in Chinese children
Source: Front Endocrinol (Lausanne). 2022 Dec 22;13:1037289. doi: 10.3389/fendo.2022.1037289 (PMC9813493; doi:10.3389/fendo.2022.1037289)
Supplement: Supplementary file 1 [file Table_1.docx]

Supplemental Table 1. Statistical packages used in this work

| No. | Statistical models | Aim | Functions | Packages |
| --- | --- | --- | --- | --- |
| 1 | Shapiro Wilk test | To evaluate the normal distribution of variables | shapiro.test | stats |
| 2 | Kruskal Wallis test | To test the statistical difference of non-normal distributed data among three groups | kruskal.test | stats |
| 3 | Hypergeometric distribution | Metabolic pathway enrichment analysis | phyper | stats |
| 4 | (O) PLS-DA | To perform supervised multivariate analysis of metabolomics data (i.e., PLS-DA and OPLS-DA model) | opls | ropls |
| 5 | PLS | To assess the relationship between Age and metabolites | opls | ropls |
| 6 | Spearman correlation | To evaluate the correlations between metabolites and age, disease duration etc. | rorr | Hmisc |
| 7 | pheatmap | To perform clustering analysis of metabolites | pheatmap | pheatmap |

Supplemental Table 2. Differential metabolites between T1D versus HC

Univariate Statistical analysis of data with or without age and gender (using logistic regression).

FC= fold change (T1D / HC)

P value was obtained from non-parametric method i.e., Mann-Whitney test in this work

FDR = false discovery rate

PvaluewithAge.adjusted= p value adjusted with age using logistic regression model

PvaluewithGender.adjusted= p value adjusted with gender using logistic regression model

PvaluewithAge&Gender.adjusted= p value adjusted with both age and gender using logistic regression model
